# Comparative Analysis of Frameless Robotic Stereotactic Biopsy with Intraoperative Sodium Fluorescein Versus Frame-Based Stereotactic Technique

**DOI:** 10.3390/diagnostics16071033

**Published:** 2026-03-30

**Authors:** Utku Özgen, Mehmet Zeki Yıldız, Mehmet Osman Akçakaya, Talat Kırış

**Affiliations:** 1 Department of Neurosurgery, American Hospital, Istanbul 34365, Turkey; talatk@amerikanhastanesi.org; 2 Department of Neurosurgery, Bahcesehir University, Istanbul 34365, Turkey; mehmetzeki.yildiz@bau.edu.tr; 3 Department of Neurosurgery, Florence Nightingale Hospital, Istanbul 34365, Turkey; moakcakaya@gmail.com

**Keywords:** frameless stereotactic biopsy, navigation, robotic biopsy, sodium fluorescein, frame-based stereotactic biopsy

## Abstract

**Background and Objectives:** The authors aimed to describe their experience performing frameless stereotactic biopsies using an Autoguide Robotic Platform and to compare the outcomes with a frame-based stereotactic technique. We would like to emphasize the importance of this study, as it is the first in the literature to use sodium fluorescein for confirmation in intracranial biopsies taken with a Stealth Autoguide Robotic Platform. **Materials and Methods:** We retrospectively evaluated 30 patients who underwent a stereotactic intracranial biopsy between June 2018 and March 2024. The patients were divided into two groups: The robotic biopsy group (n = 15) underwent a frameless image-guided stereotactic intracranial biopsy with a Stealth Autoguide Robotic Platform and optical neuronavigation system (Stealth-Station S8, Medtronic, Minneapolis, MN, USA) using intraoperative sodium fluorescein. The frame-based (Integra, CRW, New Jersey, USA) stereotactic biopsy group (n = 15) underwent a stereotactic biopsy with the use of a stereotactic planning system (Atlas Integra Software, NJ, USA and Brainlab AG, Munich, Germany) without sodium fluorescein. Preoperative MRI and CT scans were performed in all the patients. Their external cranial anatomy was registered using either facial tracing or O-Arm (Medtronic Sofamor Danek, Inc., Memphis, TN, USA). **Results:** The robotic biopsy group demonstrated a diagnostic yield of 93.3% (14/15), while the frame-based group achieved 100% (15/15), with no significant difference (*p* = 0.609). The mean calculated tip error in the robotic biopsy group was 0.42 ± 0.19 mm (range: 0.1–0.7 mm) and the postoperative targeting accuracy in the frame-based biopsy group was 0.51 ± 0.23 mm (range: 0.2–0.9 mm), with no significant difference (*p* = 0.287). The robotic biopsy group demonstrated a significantly shorter mean surgical time (40.26 ± 6.13 vs. 52.47 ± 8.92 min, *p* = 0.002). **Conclusions:** Both frame-based and robotic-assisted stereotactic biopsy techniques achieve comparable diagnostic accuracy and targeting precision. However, a robotic biopsy significantly reduces the surgical time compared to a frame-based technique. The use of intraoperative sodium fluorescein is a valuable adjunct method for confirming that biopsy specimens are obtained from the intended target site.

## 1. Introduction

Stereotactic biopsies have been used for many years as a gold standard technique for sampling lesions in the brain that are difficult to reach, risky, and unsuitable for microsurgical resection [1,2]. Although a stereotactic biopsy is a highly accurate and effective technique, the need to fix the patient’s head with a stereotactic frame before surgery and the necessity of taking a CT (computerized tomography) to determine the correct pathway to reach the lesion increase the preoperative preparation time [3]. Patients’ discomfort and difficulties in calculating the entry point are other reported disadvantages of frame-based technique [1].

For these reasons, frameless biopsy techniques have emerged as important alternatives to framed biopsy techniques, as they can register preoperative images with navigation systems by using a patient’s facial landmarks, without the need for intraoperative imaging. This advantage can help to reduce the preoperative preparation time [4,5]. In navigation-assisted frameless biopsy techniques, intraoperative navigation systems are used to visualize the position of the biopsy needle during surgery. This allows a surgeon to see the trajectory and depth of the needle during surgery, providing guidance for accurate lesion sampling without the need for a stereotactic frame [6,7,8].

Recently, robotic platforms such as SurgiScope (Intelligent Surgical Instruments & Systems), Neuromate (Renishaw), and ROSA (Zimmer Biomet Robotics) have been used for cranial biopsies. However, their large robotic arms can extend the surgical duration due to the significant space they occupy in an operating room. For these reasons, the use of platforms that occupy less space in operating rooms and have faster setup times compared to large robotic platforms has gained importance. The Autoguide Robotic System (Medtronic, Minneapolis, USA) is a user-friendly system due to its fast setup and ergonomic design. This study aimed to describe the authors’ experiences performing frameless stereotactic biopsies using an Autoguide Robotic Platform and to compare the outcomes with a frame-based stereotactic technique.

## 2. Materials and Methods

We retrospectively evaluated 30 patients who underwent a frameless stereotactic intracranial biopsy between June 2018 and March 2024. The patients were divided into two groups: The robotic biopsy group (n = 15) underwent a frameless image-guided stereotactic intracranial biopsy with a Stealth Autoguide Robotic System and optical neuronavigation system (Stealth-Station S8, Medtronic, Minneapolis, MN, USA) with intraoperative sodium fluorescein at American Hospital (Istanbul, Türkiye). The frame-based biopsy group (n = 15) underwent a frame-based stereotactic biopsy using a stereotactic frame (Integra, CRW, New Jersey, NJ, USA) with the use of a stereotactic planning system (Atlas Integra Software, New Jersey, NJ, USA, and Brainlab AG, Munich, Germany) without sodium fluorescein at Florence Nightingale Hospital (Istanbul, Türkiye).

In the robotic biopsy group, three patients underwent a biopsy as part of LITT (Laser Interstitial Thermal Therapy) procedure. In these cases, a biopsy was performed to confirm a diagnosis that was previously suspected by examining the radiological images. Informed consent was obtained from all patients in our study according to regulations of IRB/ethics committee, and the content, details, and purpose of the study were explained to them in detail. Due to the retrospective nature of the study, approval was waived. Preoperative MRI (Magnetic Resonance Imaging) and CT scans were performed. The bone thickness was calculated before surgery by using the CT images to adjust the Midas Rex drill stopper to an appropriate length in the robotic biopsy group. The external cranial anatomy was registered using either facial tracing or an O-Arm (Medtronic Sofamor Danek, Inc., Memphis, TN, USA), and the accuracy of the Stealth-Station S8 (Medtronic, Minneapolis, MN, USA) optical navigation was verified using StealthStation Software. An O-Arm (Medtronic Sofamor Danek, Inc., Memphis, TN, USA) was used for registration of patients undergoing surgery in the prone position and where registration with classical facial landmarks was difficult. To determine the accuracy, the navigation software detects the robot’s position based on the planned entry and target points, and calculates the accuracy based on the registration created using the patient’s facial landmarks. The calculated accuracy is a derivation of the angle and distance to the target point that is defined during surgical planning and registration. In the robotic biopsy Group, the targeting accuracy was determined by using navigation software. In the frame-based biopsy group, a preoperative CT scan was performed with the frame fixated on the patient’s head and it was merged with the MRI (axial, thin sliced, non-gap continuous), which was usually performed one day before surgery or on the surgery day before frame application. The procedure was performed under local anesthesia and sedation. Following the biopsy, the targeting accuracy was assessed postoperatively by CT imaging with the stereotactic frame in place. In the robotic biopsy group, the targeting error was reported as the tip error calculated by the Stealth Navigation software (Medtronic, Minneapolis, MN, USA), which computes the vector distance between the planned target point and the actual needle tip position detected intraoperatively in real time. In the frame-based group, the targeting accuracy was assessed by comparing the preoperatively planned stereotactic X, Y, and Z coordinates with the coordinates of the achieved needle tip position measured on the postoperative CT obtained with the stereotactic frame still in place, using the same stereotactic planning software. The deviation between the planned and achieved coordinates in each axis was recorded, and the overall targeting error was calculated from these values, enabling quantitative comparison of accuracy between the two groups. An accuracy of 1 mm or less was accepted during the performance of the biopsy. All the patients in robotic biopsy group received a single 5 mg/kg intravenous bolus of SF (sodium fluorescein) (Fluorescein Novartis 100 mg/mL; BBraun Melsungen, Mistelweg, Berlin, Germany) following induction of general anesthesia. For the robotic biopsy group, the samples were examined intraoperatively under a yellow 560 filter integrated into a Kinevo 900 (Carl Zeiss Meditec, Oberkochen Germany) prior to the intraoperative frozen section assessment to determine if the samples were lesional or non-lesional. The concordance between the intraoperative frozen section diagnosis and the final diagnosis from the permanent paraffin-embedded sections was prospectively recorded and calculated as a percentage of cases in which the two diagnoses were in agreement, among all the cases in which tissue was submitted for formal pathological evaluation. All the samples were then sent for routine histopathology assessment. The pathologists involved were blinded to the SF status of the samples and a definitive diagnosis was made according to most recent WHO (World Health Organization) classification. The target point was systematically planned inside the contrast-enhancing area of the lesion. All the patients were discharged home on the same day or the next morning.

## 3. Results

### 3.1. Patient Demographics

A total of 30 patients underwent a stereotactic biopsy. The robotic biopsy group consisted of 15 patients (12 males [80%], three females [20%]) with a mean age of 57.27 ± 21.89 years (median: 54 years; range: 22–91 years; IQR: 39–78 years; 95% CI: 45.14–69.39 years). The age distribution was normal (Shapiro–Wilk test: W = 0.961, *p* = 0.714). The frame-based biopsy group consisted of 15 patients (nine males [60%], six females [40%]) with a mean age of 53.87 ± 16.34 years (range: 12–82 years). There was no significant difference in age between the groups (*p* = 0.633, independent *t*-test). The gender distribution did not differ significantly (*p* = 0.231, Fisher’s exact test) (Table 1).

**Table 1:** Demographics, procedural outcomes and histopathological diagnoses.

	Robotic Biopsy Group (n = 15)	Frame-Based Biopsy Group (n = 15)	*p*-Value
**Demographics**			
Mean age (years)	57.27 ± 21.89	53.87 ± 16.34	0.633
Male/female	12/3 (80%/20%)	9/6 (60%/40%)	0.231
**Procedural Outcomes**			
Surgical time (min)	40.26 ± 6.13	52.47 ± 8.92	0.002 *
Targeting accuracy (mm)	0.42 ± 0.19	0.51 ± 0.23	0.287
Mean target depth (mm)	65.75 ± 17.18	62.35 ± 12.5	0.573
Diagnostic yield	93.3% (14/15)	100% (15/15)	0.609
**Complications**			
Asymptomatic hemorrhage	1 (6.7%)	0 (0%)	1.000
**Diagnosis**			
Glioblastoma (GBM)	6 (40.0%)	5 (33.3%)	-
High-grade glioma	2 (13.3%)	0 (0%)	-
Low-grade glioma	0 (0%)	2 (13.3%)	-
Lymphoma	3 (20.0%)	2 (13.3%)	-
Diffuse midline glioma	1 (6.7%)	0 (0%)	-
Anaplastic astrocytoma	0 (0%)	2 (13.3%)	-
Diffuse astrocytoma	0 (0%)	1 (6.7%)	-
Germinoma	0 (0%)	1 (6.7%)	-
Metastasis	1 (6.7%)	1 (6.7%)	-
Cerebritis	1 (6.7%)	0 (0%)	-
Tumefactive multiple sclerosis	0 (0%)	1 (6.7%)	
Non-diagnostic	1 (6.7%)	0 (0%)	-

* statistically significant.

### 3.2. Targeting Accuracy

In the robotic biopsy group, the mean calculated tip error measured by the navigation software was 0.42 ± 0.19 mm (median: 0.42 mm; range: 0.1–0.7 mm; 95% CI: 0.32–0.53 mm). The tip error distribution was normal (Shapiro–Wilk test: W = 0.967, *p* = 0.809). In the frame-based biopsy group, the targeting accuracy was assessed by postoperative CT imaging with the stereotactic frame in place. The mean targeting error was 0.51 ± 0.23 mm (median: 0.50 mm; range: 0.2–0.9 mm; 95% CI: 0.38–0.64 mm). There was no significant difference in the targeting accuracy between the groups (*p* = 0.287, independent *t*-test).

The mean target depth in the robotic biopsy group was 65.75 ± 17.18 mm and 58.67 ± 15.11 mm in the frame-based biopsy group. All patients achieved successful needle placement at the intended target with no significant deviation requiring trajectory adjustment.

### 3.3. Procedural Times

The mean surgical time from skin incision to wound closure was 40.26 ± 6.13 min (median: 40 min; range: 38–55 min; 95% CI: 36.87–43.65 min) in the robotic biopsy group and 52.47 ± 8.92 min (median: 51 min; range: 42–68 min; 95% CI: 47.52–57.42 min) in the frame-based biopsy group. The robotic biopsy group demonstrated a significantly shorter surgical time compared to the frame-based biopsy group (*p* = 0.002, independent *t*-test), representing an approximately 23% reduction in the operative time.

### 3.4. Diagnostic Yield

**Robotic biopsy group:** The diagnostic yield was 93.3% (14/15 patients; 95% CI: 70.2–98.8%). The histopathological examination revealed glioblastoma (GBM, IDH wild type) in six cases (40.0%; 95% CI: 19.8–64.3%), high-grade glioma in two cases (13.3%; 95% CI: 3.7–37.9%), lymphoma in three cases (20.0%; 95% CI: 7.0–45.2%), diffuse midline glioma in one case (6.7%; 95% CI: 1.2–29.8%), metastasis in one case (6.7%; 95% CI: 1.2–29.8%), cerebritis in one case (6.7%; 95% CI: 1.2–29.8%), and abscess in one case (6.7%; 95% CI: 1.2–29.8%). The single unsuccessful case was an abscess that was not sent for pathological examination but was successfully managed with antibiotic therapy. In 14 out of 15 patients, excluding the abscess that was not sent for pathological examination, the diagnosis made during the intraoperative frozen section pathological examination was consistent with the diagnosis made from the paraffin examination. The concordance rate between the intraoperative frozen section diagnosis and final permanent histopathology was 100% (14/14 evaluable cases; 95% CI: 76.8–100.0%). In the patient with cerebritis, inflammatory cells were observed in the intraoperative tissue sample, but the definitive pathology was later verified. The case was accepted as cerebritis, and the patient improved with the administered treatment. In our study, 14 out of the 15 patients exhibited contrast enhancement on the MRI, except for the patient diagnosed with an astrocytoma Grade III IDH mutant lesion, and all except one were stained intraoperatively with sodium fluorescein. The lesion that did not stain with sodium fluorescein intraoperatively was diagnosed as cerebritis. In all these cases, it was verified that the samples were taken from the lesion. SF was not used in the patient with an abscess, and despite the contrast enhancement on the imaging, no staining was observed in the patient who was later diagnosed with cerebritis following further pathological examination.

**Frame-based biopsy group:** The diagnostic yield was 100% (15/15 patients; 95% CI: 62.1–96.3%). The histopathological examination revealed glioblastoma (GBM, IDH wild type) in five cases (33.3%), followed by lymphoma in two cases (13.3%), low-grade glioma in two cases (13.3%), and anaplastic astrocytoma (WHO Grade 3) in two cases (13.3%). The single cases included diffuse astrocytoma (6.7%), germinoma (6.7%), renal cell carcinoma metastasis (6.7%), and tumefactive multiple sclerosis (6.7%). There was no significant difference in diagnostic yield between the groups (*p* = 0.609, Fisher’s exact test).

### 3.5. Complications

In the robotic biopsy group, one patient with a thalamic lesion (6.7%; 95% CI: 1.2–29.8%) experienced an intraventricular hemorrhage detected on the postoperative imaging and in the frame-based biopsy group there were no complications. In the robotic biopsy group, the follow-up imaging of the patient with the intraventricular hemorrhage demonstrated spontaneous resolution of the bleeding, and no further surgical intervention was necessary. There was no significant difference in complication rates between the groups (*p* = 1.000, Fisher’s exact test).

### 3.6. Statistical Analysis

Descriptive statistics were used to summarize the data. The continuous variables were expressed as the mean ± standard deviation, median, range (minimum–maximum), and interquartile range (IQR). The Shapiro–Wilk test was used to assess the normality of continuous variables. The continuous variables were compared between groups using an independent *t*-test for the normally distributed data and a Mann–Whitney U test for the non-normally distributed data. The categorical variables were expressed as frequencies and percentages and compared using Fisher’s exact test or a chi-square test. The Wilson score method was used to calculate the 95% confidence intervals (CIs) for the proportions. All the statistical analyses were performed using SPSS version 25.0 (IBM Corp., Armonk, NY, USA). A *p*-value < 0.05 was considered statistically significant.

### 3.7. Robotic Biopsy Surgical Technique

**Figure 1:**
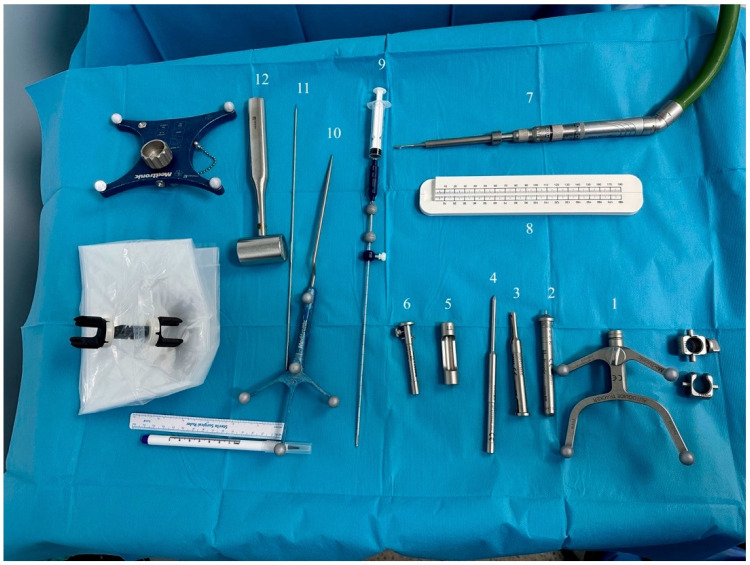
**View of surgical materials for Autoguide Surgical Platform.** The numbered components and their intraoperative functions are as follows: 1—Stealth Autoguide Tracker: An optical reference frame attached to the patient’s head for real-time intraoperative navigation registration; 2—Autoguide Height Guide: The Autoguide height guide was flipped and reinserted into the Autoguide Stealth Tracker with the round tip facing down and locked. The Autoguide positioning arm was unlocked and the patient’s scalp was touched with the round tip of the height guide; 3—Autoguide Stab Incision Drill Guide: A cannulated sleeve that channels the drill to the planned entry point through a small stab incision; 4—Autoguide Stab Incision Obturator: The Autoguide Stab Incision Obturator was inserted into the Autoguide Stab Incision Drill Guide until it made contact with the skull; 5—Autoguide Tapping Tube: A threaded sleeve driven into the outer cortex to anchor the working channel at the skull; 6—Autoguide Reducing Tube: An inner cannula that narrows the working channel to match the diameter of the biopsy needle; 7—Midas Rex Drill: A high-speed pneumatic drill used to drill through the outer cortex; 8—Measurement Tool: A depth-calibration rod used to confirm the needle insertion length against the pre-planned trajectory; 9—Autoguide Biopsy Needle with Syringe Attached: A side-cutting biopsy needle advances along the trajectory to aspirate tissue samples from the target lesion; 10—Navigation Probe: A tracked pointer used intraoperatively to verify the registration accuracy before needle insertion; 11—Kirschner Wire: A stiff guide wire used to breach the dura; 12—Surgical Hammer: Used to seat the tapping tube firmly into the outer cortical bone.

Following the induction of general anesthesia, each patient was positioned either prone or supine based on the lesion’s location, and their head was secured in a three-pin head frame. The preoperative MRI or CT images were collected and transferred into the navigation software. An Autoguide head clamp was mounted onto the patient’s head clamp. The navigation arm and Autoguide positioning arm were mounted on the Autoguide head clamp adapter. The Autoguide targeting unit was mounted on the Autoguide positioning arm and the Autoguide positioning arm was placed into the desired position and locked by using the ratchet locking mechanism on the Autoguide positioning arm. Navigation was recorded using facial tracking in most of the cases, but in one patient who was operated on in the prone position, an O-arm was used. The biopsy needle entry point, trajectory, and target depth were planned by using StealthStation software the day before surgery on the control unit and transferred to the navigation system. The operating table was prepared by opening the necessary surgical instruments in a sterile manner (Figure 1).

**Figure 2:**
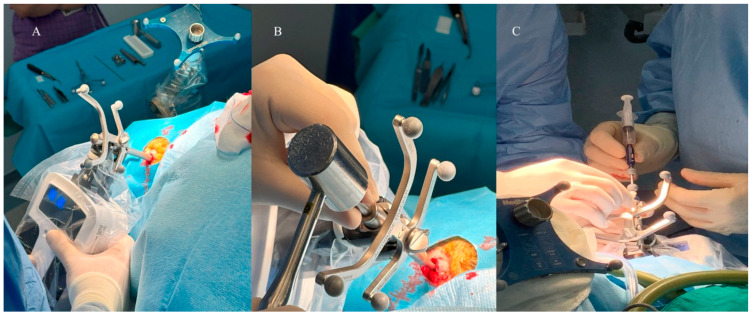
(**A**) Intraoperative view of Autoguide Robotic Platform. The Autoguide height guide is reinserted into the Autoguide Stealth Tracker with the round tip facing down and locked. The Autoguide positioning arm is unlocked and the patient’s scalp is touched with the round tip of the height guide. (**B**) Anchoring the stab incision obturator guide at the skull. (**C**) Moving the biopsy needle to the preoperatively defined target and obtaining tissue samples by using syringe.

The patient, Autoguide targeting unit and Autoguide positioning arm were draped in a sterile fashion. The robotic system was manually repositioned by the surgeon to the planned entry point on the surface of the skin within a few centimeters and locked in place. The Autoguide Stealth Tracker was verified at the navigation reference frame. The Autoguide height guide was flipped and reinserted into the Autoguide Stealth Tracker with the round tip facing down and locked. The Autoguide positioning arm was unlocked and the patient’s scalp was touched with the round tip of the height guide (Figure 2).

When the symbols on the Autoguide targeting unit and Autoguide control unit indicated that the active surgical plan was within reach (dotted sphere touched the circle), the positioning arm was locked and the height guide was removed from the tracker. By using the Autoguide control unit, the Autoguide targeting device was positioned automatically according to the previously structured plan on the navigation system.

**Figure 3:**
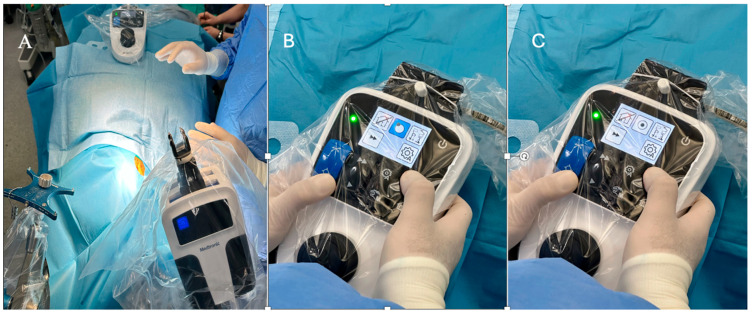
Intraoperative view of Autoguide positioning arm and control unit. (**A**) Autoguide positioning arm is adjusted to home position and locked by using ratchet locking mechanism on Autoguide positioning arm. (**B**) Autoguide control unit indicate that active surgical plan is within reach (dotted sphere touches circle). (**C**) Autoguide control unit is checked and verified that dotted sphere is inside circle.

The Autoguide control and targeting unit were adjusted so that the dotted sphere was positioned inside the circle, as shown in Figure 3. After aligning with the active surgical plan, any deviation from the target point was detected on the navigation screen. Deviations of 1 mm or less were accepted in all cases.

**Figure 4:**
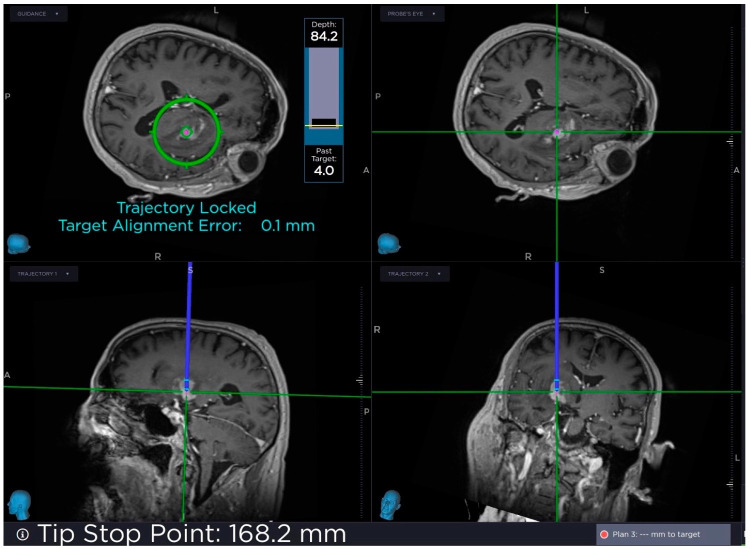
Heads-up display on StealthStation monitor of real-time navigation information. Tip stop point, target alignment error and target depth are shown.

A skin incision was made and the Autoguide Stab Incision Drill Guide was inserted into the Stealth Autoguide Tracker and tightened by using the thumb screw on the Stealth Autoguide Tracker. The Autoguide Stab Incision Obturator was inserted into the Autoguide Stab Incision Drill Guide until it made contact with the skull. By using a surgical hammer, the Stab Incision Obturator Guide was anchored at the skull. The Autoguide Stab Incision Drill Guide was unlocked and lowered until it made contact with the skull. An Autoguide tapping tube was slid over the Autoguide, then the Stab Incision Obturator and the drill guide were hammered at the skull. The target alignment error was checked on the Stealth Station screen to determine whether the error was still within an acceptable range after hammering to anchor the drill guide (Figure 4). A Midas Rex drill was inserted into the drill guide and stopper of the drill was adjusted according to the thickness of the skull.

**Figure 5:**
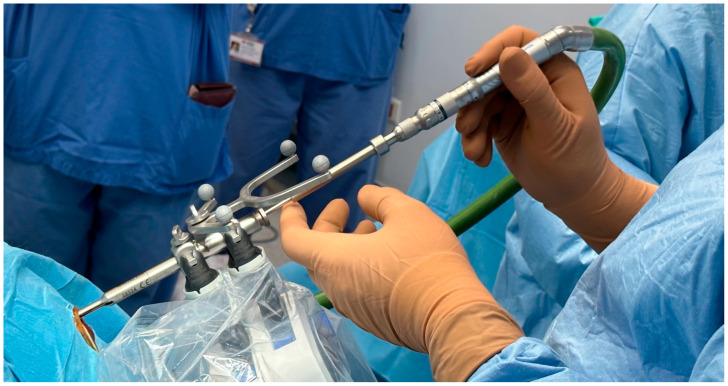
Intraoperative view of Midas Rex drill, which is inserted in the drill guide and the stopper of the drill is adjusted according to the thickness of the skull.

**Figure 6:**
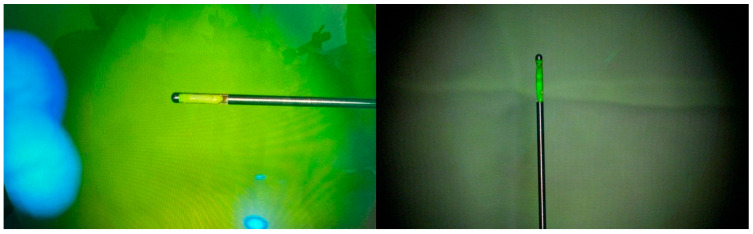
Intraoperative microscopic view of biopsy specimens stained with sodium fluorescein. Positive fluorescein staining—bright yellow-green fluorescence under blue-light excitation (excitation wavelength: 460–500 nm)—confirms that the specimen was obtained from the enhancing lesion, where blood–brain barrier disruption permits fluorescein extravasation.

The preoperative CT scans were used to determine the thickness of the bone. The skull was drilled (Figure 5) and the dura was breached by using a Kirschner wire. The Autoguide reducing tube was inserted and the biopsy needle was inserted into the reducing tube. The tip point and target alignment error were checked by using Stealth Navigation Software. The samples were examined under a microscope’s yellow 560 filter after administering sodium fluorescein approximately 15 min before the procedure, and it was confirmed that the biopsy was performed on the correct target (Figure 6). Furthermore, the intraoperative pathologic examination confirmed that the target was accurate.

### 3.8. Frame-Based Stereotactic Biopsy Technique

Following induction of general anesthesia, each patient was positioned supine and a Leksell stereotactic frame (Integra, CRW, NJ, USA) was applied to their skull using four fixation pins. After the frame application, fiducial markers were attached to the frame and the patient underwent CT imaging. The CT images were used to establish a three-dimensional coordinate system relative to the frame. The preoperative MRI images were fused with the CT images using navigation software (Brainlab AG, Munich, Germany) to optimize target localization. The CT images obtained after the stereotactic frame application provided the coordinate reference system, while the MRI images offered superior lesion visualization and anatomical detail.

**Figure 7:**
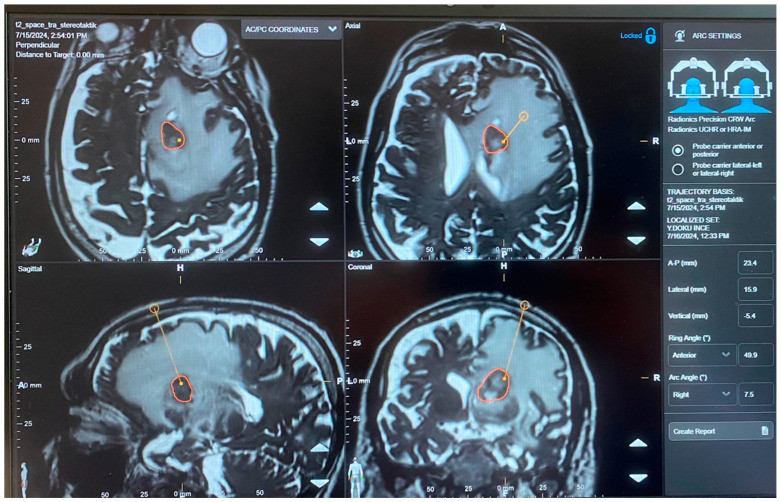
Stereotactic trajectory planning using Brainlab AG, Munich, Germany navigation software. The target lesion is outlined in orange with the planned trajectory (yellow line) shown in all three orthogonal planes. The right panel displays the trajectory coordinates, including the anterior–posterior (A-P), lateral, and vertical measurements, along with the ring and arc angle settings for frame-based stereotactic guidance. The software allows for precise three-dimensional localization and optimal trajectory selection to minimize neurovascular structures along the approach.

The target coordinates were calculated using the stereotactic planning software based on the lesion’s location on the CT imaging (Figure 7).

The target was selected within contrast-enhancing regions when present, or based on T2/FLAIR hyperintensity for non-enhancing lesions. The entry point coordinates were calculated to provide the safest trajectory, avoiding the eloquent cortex, sulci, and major vascular structures.

**Figure 8:**
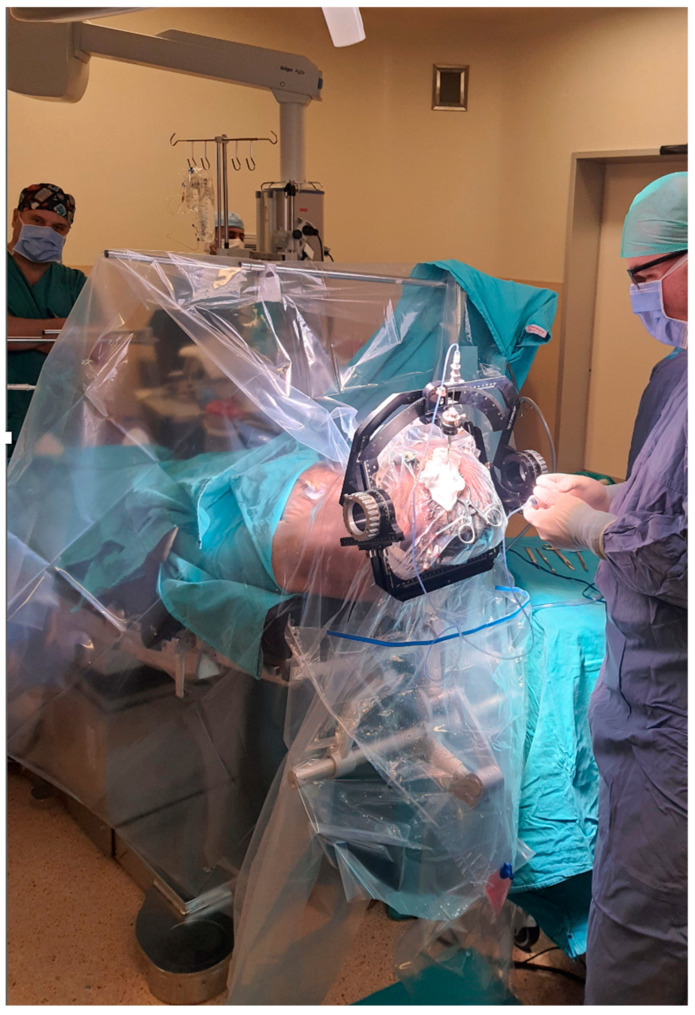
Intraoperative view of frame-based stereotactic brain biopsy procedure. The stereotactic frame is secured to the patient’s head, providing a rigid reference system for precise target localization. The surgical team operates under standard neurosurgical lighting with stereotactic guidance for accurate needle trajectory planning and execution.

Each patient was positioned on the operating table with the stereotactic frame secured to the table adapter (Figure 8).

Using the calculated X, Y, and Z coordinates, the trajectory guide arc was mounted onto the stereotactic frame and adjusted to the predetermined coordinates. The entry site on the scalp was identified using the trajectory guide, and local anesthetic with epinephrine was infiltrated.

A small skin incision (approximately 3 cm) was made at the entry point, and a high-speed drill (Medtronic, Minneapolis, MN, USA) was used to create a burr hole. The dura was coagulated and opened with a sharp needle. A side-cutting biopsy needle was introduced through the trajectory guide cannula and advanced to the calculated target.

Multiple tissue samples were obtained at the target level (at least four samples were obtained—if needed, the needle was placed 1 mm above or below the target according to the target lesion for four more additional samples) and sent for frozen section examination and permanent histopathology. In all cases, immediate postoperative CT imaging was performed with the stereotactic frame still in place to measure the actual needle trajectory and calculate the targeting error by comparing the achieved coordinates with the planned coordinates. The targeting error was quantified by comparing the preoperatively planned stereotactic target coordinates (X, Y, Z) with the coordinates of the actual needle tip position measured on the postoperative CT acquired with the stereotactic frame still in situ, using the stereotactic planning software. The deviation between the planned and achieved coordinates across all three spatial axes was recorded for each case, yielding a quantitative targeting error value directly comparable to the tip error metric generated by the navigation software in the robotic biopsy group. An accuracy of 1 mm or less was accepted when performing the biopsy. The stereotactic frame was then removed, and the pin sites were dressed.

## 4. Discussion

Many studies have been published in the literature regarding the reliability and usefulness of brain biopsy with stereotactic techniques [5,9,10]. Instead of the freehand biopsy technique, frame-based and frameless stereotactic and robot-assisted biopsy techniques have become more common today due to their increased safety and accuracy. With the advancement of robotic technology, frameless biopsy techniques have become more prominent for stereotactic biopsies compared to frame-based techniques [1,11,12]. Although frame-based techniques are considered the gold standard for stereotactic biopsy, frameless biopsy techniques have begun to be preferred, due to reasons such as the need for imaging after placing the frame, negatively impacting patient comfort, and prolonged surgical time [1]. Some of the most important advantages of robotic surgery compared to frame-based stereotactic procedures include the automatic positioning of the robotic platform to the target, which helps prevent errors that may arise due to manually controlling the tracking device and trajectory guide [13]. The Autoguide Robotic Platform is a suitable option for an intracranial biopsy due to its small robotic arm, which occupies much less space in an operating room compared to similar robots, and its high accuracy and precision. The Autoguide Robotic Platform is a system with high maneuverability that can quickly respond to changes in the navigation plan and has a short learning curve. While the Autoguide platform has a shorter learning curve compared to larger robotic systems, institutions that adopt this technology should anticipate an initial adaptation period during which the procedural times and targeting precision may be suboptimal. Mallereau et al.’s matched cohort analysis comparing frame-based and robot-assisted stereotactic brain biopsies demonstrated comparable diagnostic yields and safety profiles between the two approaches, supporting the feasibility of transitioning to robotic platforms without compromising patient outcomes during the learning phase [14]. Unlike other robotic devices which have larger robotic arms, it does not require adjusting the robot to a reference point before making changes to a plan [3].

The most significant finding of our comparative analysis is the substantial difference in the procedural efficiency between the two techniques. The robotic biopsy group demonstrated a significantly shorter mean surgical time (40.26 ± 6.13 vs. 52.47 ± 8.92 min, *p* = 0.002), representing an approximately 23% reduction in operative time. Consistent with our findings, Dhawan et al.’s meta-analysis of 15 studies involving 2400 patients found no significant difference in the diagnostic yields between frame-based and frameless biopsy groups, but significantly shorter surgical times for the frameless group [6]. These time differences reflect fundamental procedural distinctions between the two techniques. A frame-based stereotactic biopsy requires several sequential steps that contribute to a prolonged operative time [12]. Following induction of general anesthesia, the stereotactic frame must be rigidly fixed to the skull and after the frame’s application, the patient must undergo CT imaging with the stereotactic frame to establish the three-dimensional coordinate system. Subsequently, the target coordinates must be manually calculated, the entry points determined, and the trajectory arc manually adjusted to align with the calculated X, Y, and Z coordinates [15]. Each of these steps is sequential and time-dependent, with no opportunity for a parallel workflow. In contrast, the Autoguide Robotic Platform features automatic positioning to the target, eliminating errors that may arise from manually controlling the trajectory guides in frame-based procedures [13]. The small robotic arm occupies minimal operating room space compared to larger robotic systems, and its ergonomic design facilitates rapid setup and workflow. Unlike other robotic devices with larger arms, the Autoguide system does not require adjusting to a reference point before making trajectory changes, contributing to its efficiency and short learning curve [3]. The mean surgical time for an intracranial biopsy varies between 30 and 127 min in the literature [13,16,17]. In our study, the mean surgical time was 40.26 ± 5.92 (range: 38–55 min). The use of a small incision during surgery, the robot’s ability to quickly and automatically position itself according to the navigation plan, and the real-time tracking of the biopsy needle’s correct position during navigation can be cited as factors that contribute to shortening the surgery.

Postoperative hemorrhage is one of the significant complications of stereotactic biopsy, and it has been reported in the literature to occur in 2% to 5% of cases. It can be asymptomatic in 20% to 25% of patients [18,19]. In the robotic biopsy group, one patient with a thalamic lesion (6.7%; 95% CI: 1.2–29.8%) experienced an intraventricular hemorrhage detected on the postoperative imaging and in the frame-based biopsy group one patient (6.7%; 95% CI: 1.2–29.8%) experienced an asymptomatic intralesional hemorrhage detected on the postoperative imaging. This complication was asymptomatic and required no additional treatment or intervention. The 6.7% hemorrhagic complication rate observed in the robotic group, while falling within the generally reported range of 2–5% for stereotactic biopsy, warrants further consideration. The single hemorrhagic event occurred in a thalamic lesion—a location associated with elevated vascular risk due to the proximity of perforating vessels. This underscores the importance of meticulous trajectory planning to avoid vascular structures, a task facilitated by the real-time navigation feedback from the Autoguide platform. Notably, Mallereau et al.’s large prospective series comparing robotic and image-guided surgery systems reported comparable complication profiles between the two approaches, suggesting that hemorrhagic risk is largely inherent to the biopsy procedure and lesion location rather than to the robotic platform itself [20].

In the robotic biopsy group, we did not postoperatively determine the accuracy through a secondary method because our diagnostic biopsy rate was 93.3%, indicating that the robot was accurate in comparison to the preoperative plans based on neuroradiological imaging. As we considered one lesion to be an abscess based solely on the preoperative imaging and clinical findings, the sample obtained from this one patient was sent for microbiological examination instead of pathological analysis; therefore, this patient was not included in our calculation of the diagnostic biopsy rate. Following the microbiological examination, the diagnosis of abscess was confirmed, and after the appropriate antibiotic treatment, the follow-up imaging showed near-complete regression of the lesion and improvement in the patient’s symptoms.

In the study published by Kreatsoulas et al., which represents one of the largest series in the literature involving biopsies performed using an Autoguide Robotic Platform, a diagnostic biopsy rate of 100% was reported. The authors emphasized that postoperative imaging is not required to confirm diagnostic accuracy and is not considered cost-effective [3]. It should be noted that it is not possible to determine true accuracy in real time without performing an intraoperative MRI or CT scan with the biopsy cannula in place. However, in clinical practice with an Autoguide Robotic Platform, we believe obtaining intraoperative imaging is not required and is significantly more costly for patients. In the robotic biopsy group, we tracked the real-time position of the biopsy needle using the navigation software during the surgery and based our calculations on the needle tip error value computed by the navigation system. In the robotic biopsy group, intraoperative imaging was used for one patient. A biopsy was taken from a patient with a left temporal lesion in the prone position by using the O-arm.

In the literature, the failure rate of stereotactic intracranial biopsies ranges from 2% to 10%, and it is an important issue to address as it necessitates repeat biopsies [8,21,22]. Fluorescent dyes, such as 5-aminolevulinic acid, indocyanine green, and sodium fluorescein, are substances used in microsurgery and stereotactic biopsies [23,24,25]. In our study, for the lesions that were partially or entirely contrast enhancing, the contrast-enhancing part of the lesion was targeted, and a biopsy was taken using the Autoguide Robotic Platform. The biopsy specimen was then examined under a microscope with a yellow 560 filter, and the staining with sodium fluorescein confirmed that the biopsy was taken from the appropriate location. In their study, Thien and his colleagues reported that the use of sodium fluorescein had a positive predictive value of 100% and a negative predictive value of 25% for confirming the biopsy specimen when using a stereotactic frame [26]. Mallereau reported that the inconclusive biopsy range is 2.6–11% for frameless biopsies and 0.7–15.7% for frame-based interventions. Atai published a retrospective review of 75 intracranial biopsy cases using an Autoguide Robotic Platform and diagnostic tissue was obtained in 100% of cases [27]. In 14 out of 15 patients in this study, the intraoperative frozen section pathological examination revealed that all samples were taken from the preoperatively desired target. The lesion which was defined as an abscess was not sent to pathology, but the samples were cultured for defining the microorganism and deciding the suitable medical therapy. Our diagnostic yield was 93.3%. In our study, 13 out of 15 specimens were stained with sodium fluorescein intraoperatively. The pathology of one of the lesion that did not stain with sodium fluorescein was determined to be cerebritis, and we could also not stain the lesion that was defined as an abscess with sodium fluorescein because of its liquid nature. We would like to emphasize the importance of this study as it is the first in the literature to use sodium fluorescein for confirmation in intracranial biopsies taken with an Autoguide robot and to demonstrate that it improves biopsy accuracy. We believe that using sodium fluorescein staining is essential after a robotic biopsy in order to increase the diagnostic yield and to confirm that the biopsy was taken from the correct target. A recent systematic review by Bianconi et al. confirmed that fluorescence-guided surgery with sodium fluorescein, 5-ALA, and ICG demonstrates variable but promising efficacy across different tumor types, with sodium fluorescein showing particular utility in high-grade lesions exhibiting blood–brain barrier disruption [28]. This supports our approach of employing sodium fluorescein as an intraoperative adjunct to confirm adequate tissue sampling from the targeted lesion.

Our study demonstrates that although frame-based stereotactic biopsy has been accepted as the gold standard in the literature for years, robotic-assisted biopsy, augmented with intraoperative sodium fluorescein, represents a safe and reliable alternative method with a comparable diagnostic yield, targeting accuracy, and complication rates. Its significantly shorter surgical time and preoperative preparation time, while maintaining similar efficacy, constitute important advantages. The diagnostic challenges inherent to minimally invasive biopsy are not unique to intracranial lesions; spinal cord tumors present with equally demanding targets due to the eloquence of surrounding neural tissue and limited access. Mallereau et al. recently reported a multicenter series of 61 spinal cord biopsy cases, providing practical technique-specific guidance and identifying the key determinants of diagnostic success and complication avoidance in this high-risk procedure [29]. These findings further reinforce the value of meticulous planning and intraoperative confirmation strategies across all stereotactic biopsy procedures.

This study has several limitations that warrant acknowledgment. First, this is a two-center, non-randomized retrospective study: the robotic biopsy group was operated on at American Hospital Istanbul under general anesthesia by a single surgeon using a Stealth Autoguide Platform, while the frame-based group was operated on at Florence Nightingale Hospital under local anesthesia and sedation by a different surgical team using a Leksell frame system. The institutional differences in anesthetic protocol, surgical team experience, patient work-up pathways, and operating room setup constitute sources of potential selection bias and confounding that limit the validity of direct between-group comparisons. The patient selection criteria were not prospectively harmonized across the centers, and systematic differences in lesion characteristics, referral patterns, or imaging protocols between the two hospitals may have independently influenced outcomes, such as the diagnostic yield, complication rate, and procedural time. These inter-institutional variables cannot be fully controlled for in a retrospective design and represent the most important structural limitation of the current study. Second, and most critically, intraoperative sodium fluorescein was used exclusively in the robotic biopsy group and was not available in the frame-based group due to differing institutional protocols. Sodium fluorescein therefore functions as a confounding variable in any direct comparison of diagnostic outcomes between the two techniques: it is impossible to determine from the current data whether any differences in the diagnostic success are attributable to the robotic platform itself, to the fluorescence guidance, or to their combination. The contribution of sodium fluorescein to the 93.3% diagnostic yield of the robotic biopsy group cannot be quantified separately from the effect of the robotic platform, and readers should exercise caution when interpreting the between-group differences in the diagnostic yield in this context. A rigorous evaluation of sodium fluorescein as an adjunct to stereotactic biopsy would require a controlled design in which fluorescein use is randomized or held constant across technique groups. Third, the sample size of 15 patients per group limits the statistical power for detecting small but clinically meaningful differences in the diagnostic yield or complication rates. Fourth, the retrospective design precludes standardization of tissue sampling protocols and postoperative imaging timing across all cases. Prospective randomized trials with larger, multicenter cohorts, harmonized protocols, fluorescein-controlled comparison arms, and comprehensive cost-effectiveness analyses are required to definitively establish the superiority or equivalence of robotic versus frame-based stereotactic biopsy.

## 5. Conclusions

Robotic-assisted and frame-based stereotactic biopsy techniques achieved comparable targeting accuracy, diagnostic accuracy, and safety profiles with similar complication rates. However, the robotic biopsy group demonstrated a significantly shorter surgical time compared to the frame-based biopsy group. The automated positioning capabilities of the robotic platform contributed to improved procedural efficiency and reduced anesthesia exposure. The use of intraoperative sodium fluorescein is a valuable adjunct technique for confirming that biopsy specimens are obtained from the intended target lesion. Both techniques represent safe and effective options for sampling deep-seated intracranial lesions, with technique selection guided by institutional resources and surgeon experience.

## Data Availability

Study data is unavailable due to privacy and ethical restrictions of our hospital.

## Abbreviations

CT: Computerized Tomography; MRI: Magnetic Resonance Imaging; LITT: Laser Interstitial Thermal Therapy
